# Stick, partial slip and sliding in the plane strain micro contact of two elastic bodies

**DOI:** 10.1098/rsos.140363

**Published:** 2014-11-12

**Authors:** George G. Adams

**Affiliations:** Department of Mechanical and Industrial Engineering, Northeastern University, Boston, MA 02115, USA

**Keywords:** friction, adhesion, slip

## Abstract

The plane strain problem of a curved elastic body pressed against an elastic half-space is considered. The effect of adhesion is included through the use of surface energy in a manner similar to the well-known JKR theory for spherical contacts. The compressive normal force is held constant while a tangential force is gradually increased from zero. The contact is characterized by complete stick up to a critical value of the tangential force when there is a transition either directly to complete sliding or to a partial slip state in which a central stick region is surrounded by two slip regions. In the latter case, at a finite value of the stick zone width, a second critical condition exists at which there is a transition from partial slip to complete sliding. This behaviour is determined for a range of dimensionless values of the work of adhesion, the assumed constant shear stress during slip/sliding and the initial compressive load.

## 2. Introduction

When a curved elastic body is pushed against a flat elastic body of either an elastically similar or dissimilar material, a contact region is formed whose dimensions can often be found from the well-known Hertz equations if the effects of friction and adhesion can be neglected. If the two materials are elastically similar, then the normal contact stresses do not produce relative tangential displacements and hence the inclusion of friction does not affect the result. Furthermore, the subsequent application of a tangential force does not produce relative normal displacements and so the contact is again unaffected. Even when the materials are elastically dissimilar, the effect of a tangential/normal load on relative normal/tangential displacements is generally small enough to be neglected [1].

With this coupling neglected, Cattaneo [2] and later Mindlin [3] showed that complete stick necessitates an infinite shear stress at the boundary of the contact and consequently is not possible without adhesion. The amount of relative tangential displacement induced by the tangential force depends upon the assumed friction relation. Under the assumption of Amontons–Coulomb friction, it was shown that for any value of the tangential load below that which is necessary for complete sliding, a finite-width central stick zone exists which is surrounded by region(s) of slip. This type of behaviour is true for both the three-dimensional case of a sphere contacting a half-space as well as for the two-dimensional plane strain case of a cylinder contacting a half-space. It is noted that in the former case, the contact area and the resulting slip and stick zone boundaries remain circular. In each geometry, as the tangential force is increased the stick zone shrinks and eventually vanishes, at which point complete sliding commences.

The effect of adhesion, which tends to be important at the micro- and nanoscales, is to increase the contact area beyond that which is predicted by Hertz. The JKR theory [4] for spherical contacts treats the effect of adhesion by including the surface energy. The resulting stresses are singular at the contact boundary which is similar to the behaviour of a crack tip in linear elastic fracture mechanics. In contrast to the surface energy approach, the DMT theory [5] is equivalent to including the force of adhesion which acts outside of the contact region as an added term to the applied force. Thus, the stress distribution and displacement profiles are the same as with Hertz, but with a greater equivalent applied load. It was shown by Muller *et al.* [6] that the reason that these two theories produce somewhat different results is that each is valid in a different limit of the Tabor parameter (*μ*) defined as [7]

2.1 $$\mu ={\left(\frac{Rw^{2}}{E^{*2}Z_{0}^{3}}\right)}^{1/3},$$

where *R* is the radius of curvature, *w* is the work of adhesion, E* is the composite modulus (defined later) and *Z*_0_ is the equilibrium spacing of two half-spaces of the contacting materials. Qualitatively, the Tabor parameter represents the ratio of the elastic deformation to the range of adhesive forces. A theory which is valid for a range of the Tabor parameter is given by Maugis [8], using a simple analytical approximation of the adhesive stresses, which allows for closed form results to be obtained. A plane strain version of the Maugis theory also exists [9], and the JKR limit is investigated by Johnson & Greenwood [10]. All of these analyses [4–10] neglect friction or, equivalently, neglect the coupling between normal/shear stresses and relative tangential/normal displacements.

The contact, with adhesion, of a sphere and a half-space in which there is both normal and tangential loading was considered by Savkoor & Briggs [11] and Johnson [12]. The JKR theory was modified to allow a tangential as well as a normal force. The breaking of complete stick now occurs at a finite value of the tangential load. In calculating this value, it was necessary to average the critical mode II and mode III stress intensity factors over the periphery of the contact. It is only through the stress intensity factor that the coupling effect is included. A complicating factor is that the work of adhesion can vary with mode-mixity (e.g. [13]). After complete stick is broken, the Maugis theory is used for the normal loading and a constant shear stress condition is used for the slip zone. The assumption of constant shear stress, rather than Amontons–Coulomb friction, is considered valid for nanoscale contacts [12]. However, the region in which tangential forces act is not precisely defined and so some simplifying assumptions needed to be made.

Sari *et al.* [14] considered the effect of adhesion on the contact area in the plane strain version of the Cattaneo/Mindlin problem. The contact area is determined by the adhesive forces using the Maugis theory without regard to the tangential forces. The tangential forces are distributed between a central stick region and the surrounding slip zones in which the shear stress is assumed constant.

In the case of a carbon nanotube (CNT) adhering to a silicon substrate, it has been experimentally observed by Whittaker *et al.* [15] that the force needed to initiate sliding is independent of the contact width up to widths of approximately 230 nm. In that configuration, the direction of slip is along the axis of the cylindrical tube, rather than perpendicular to the axis as is the case in the previously cited investigations. Wu *et al.* [16] presented an analysis of such a nanoscale contact. Adhesion prevents relative slip of the CNT along the substrate until a critical load is reached at which adhesion between the CNT and the corner of the substrate is broken. Nonetheless, a subsequent increase in loading can be accommodated by a combination of shear stress in the slip region and adhesion at the boundary between the stick and slip zones. The results of this analysis were able to predict the experimentally observed ‘contact-length independent slip’ [15].

The coupling effect between normal/shear stresses and relative tangential/normal displacements is included in the axisymmetric Hertz problem by Spence [17] and for the plane strain case by Zhupanska & Ulitko [18]. Coupling was also introduced into the plane strain generalized JKR adhesion model of a cylinder on a half-plane by Chen & Gao [19]. In that investigation, the emphasis was on the effect of cylinder mismatch, which is particularly relevant to biological applications in cell adhesion. The results of Spence [17], Zhupanska & Ulitko [18] and Chen & Gao [19] (without cylinder mismatch) showed small differences with the corresponding uncoupled analyses.

In this paper, a plane strain analysis is presented of slip of an elastic cylinder on an elastic half-space with adhesion. The normal force is kept constant as the tangential force is increased from zero. The coupling effect is included only insofar as in the inclusion of mode I and mode II stress intensity factors. As in the three-dimensional problem [11,12], the breaking of the complete stick contact is first determined. That process is somewhat more clear-cut in this two-dimensional analysis because the shear loading is only mode II. Nonetheless, the issue of the effect of mode-mixity on the work of adhesion [13] is still a complicating factor.

After full-stick is broken, two distinct possibilities exist. The contact may transition to complete sliding with a constant shear stress and with the contact area now determined by the Hertz equation. The other possibility is the existence of a central stick zone surrounded by two slip zones, each with constant shear stress. In that case, the contact region is also determined from the Hertz equations (adhesion has been broken at the contact boundary) but the stick–slip boundary is nonetheless determined by an adhesion condition which is strictly mode II because the normal stresses at this boundary are bounded.

## 3. Mathematical formulation

**Figure 1.**
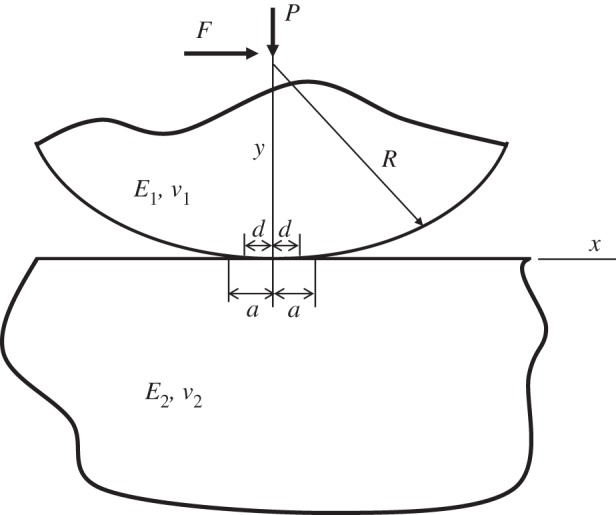
An elastic cylinder pressed against an elastic half-space by a force per unit depth (*P*) and acted upon by a tangential force per unit depth (*F*) in the presence of adhesion. The contact width is 2*a* and the stick zone width is 2*d*.

Consider the plane strain elasticity problem of a cylinder pushed against a flat elastic half-space by a force per unit depth *P* in the presence of adhesion. Alternatively, the cylinder may represent an asperity on the surface of another elastic half-space. A tangential force per unit depth *F* will be applied later as shown in figure 1. The resulting contact half-width during normal and/or tangential loading is denoted by *a*. The cylinder/asperity of radius of curvature *R* and the half-space have Young’s elastic moduli given by *E*_1_ and *E*_2_ and Poisson’s ratios of *ν*_1_ and *ν*_2_, respectively. The effect of adhesion is modelled using the work of adhesion (*w*) in a manner similar to the well-known JKR model [4] of a spherical contact subjected to normal loading.

### 3.1 Normal loading only

The plane strain version of the JKR model [11,12] can be considered as the superposition of the Hertz solution, with contact pressure *p*_1_(*x*) and force per unit depth *P*_1_:

3.1 $$p_{1}(x)=\frac{2P_{1}}{\pi a_{0}^{2}}\sqrt{a_{0}^{2}-x^{2}},\;-a_{0}<x<a_{0},$$

where

3.2 $$P_{1}=\frac{\pi E^{*}a_{0}^{2}}{4R}\;\text{and}\;\frac{1}{E^{*}}=\frac{1-\nu_{1}^{2}}{E_{1}}+\frac{1-\nu_{2}^{2}}{E_{2}}$$

with effective elastic modulus (E*), and that of a flat-ended punch with corresponding values of *p*_2_(*x*) and *P*_2_, i.e.

3.3 $$p_{2}(x)=\frac{P_{2}}{\pi \sqrt{a_{0}^{2}-x^{2}}},$$

where *a*_0_ is the value of *a* in the absence of a tangential force. The value of *P*_2_ is found from the critical value of the mode I stress intensity factor, i.e.

3.4 $$K_{I}=-\lim_{x\to a}\sqrt{2\pi (a_{0}-x)}p(x)=\frac{P_{2}}{\sqrt{\pi a_{0}}}=-\sqrt{2wE^{*}},\Rightarrow P_{2}=-\sqrt{2\pi wE^{*}a_{0}}.$$

Thus, the total normal force (*P*=*P*_1_+*P*_2_) is given in dimensionless form by

3.5 $$\frac{P}{E^{*}R}=\frac{\pi }{4}{\left(\frac{a_{0}}{R}\right)}^{2}-{\left(2\pi \frac{w}{E^{*}R}\frac{a_{0}}{R}\right)}^{1/2}.$$

Note that the force *P* and its components are considered positive in compression.

The pull-off force can be obtained by setting the derivative of *P* with respect to *a*_0_ equal to zero in equation (3.5), yielding the critical contact half-width at pull-off as well as the pull-off force

3.6 $$\begin{array}{rl} {\left(\frac{a_{0}}{R}\right)}_{\mathrm{cr}} & ={\left(\frac{2w}{\pi E^{*}R}\right)}^{1/3}\\ \text{and}\;{\left(\frac{P}{E^{*}R}\right)}_{\mathrm{cr}} & =-3{\left(\frac{\pi }{16}\right)}^{1/3}{\left(\frac{w}{E^{*}R}\right)}^{2/3}≅-1.744{\left(\frac{w}{E^{*}R}\right)}^{2/3}. \end{array}\}$$

This result agrees with that of Johnson & Greenwood [10].

### 3.2 Inclusion of a tangential force

Now a tangential force per unit depth (*F*) is applied while keeping the normal force constant. The resulting shear stress distribution (*τ*) and mode II stress intensity factor (*K*_II_) are given by

3.7 $$\tau =\frac{F}{\pi \sqrt{a^{2}-x^{2}}}\;\text{and}\;K_{\mathrm{II}}=\frac{F}{\sqrt{\pi a}}.$$

The contact half-width (*a*) is now determined by a combination of mode I and mode II loading,

3.8 $$K_{I}^{2}+K_{\mathrm{II}}^{2}=\frac{F^{2}}{\pi a}+\frac{P_{2}^{2}}{\pi a}=2E^{*}w,$$

in which it has been assumed that the work of adhesion is given by its mode I value, i.e. an ‘ideally brittle’ interface [13].

Using *P*_2_=*P*−*P*_1_ where now *P*_1_=πE*a^2^/4*R* and multiplying equation (3.8) by *πa*/*P*^2^ leads to

3.9 $${\left(\frac{F}{P}\right)}^{2}+1-\frac{\pi }{2}{\left(\frac{a}{R}\right)}^{2}/\left(\frac{P}{E^{*}R}\right)+\frac{\pi^{2}}{16}{\left(\frac{a}{R}\right)}^{4}/{\left(\frac{P}{E^{*}R}\right)}^{2}=2\pi \left(\frac{a}{R}\right)\left(\frac{w}{E^{*}R}\right)/{\left(\frac{P}{E^{*}R}\right)}^{2}.$$

Summarizing, for prescribed values of the dimensionless normal force (*P*/E*R) and of the dimensionless work of adhesion (*w*/E*R), equation (3.9) relates the dimensionless contact half-width (*a*/*R*) to the ratio of tangential to normal force (*F*/*P*).

One restriction on the results from equation (3.9) is that the rigid punch part of the normal stress distribution must remain tensile (i.e. *P*_2_<0) in order to avoid overlap of the deformed surfaces in the immediate neighbourhood of the contact. It is also noted that *P*_2_=0 corresponds to Hertz contact without adhesion and it therefore corresponds to pure mode II failure. Thus, it is required that

3.10 $$\frac{a}{R}\geq \sqrt{\frac{4}{\pi }\frac{P}{E^{*}R}}.$$

It is noted that from equation (3.9) that *P*_2_=0 corresponds to

3.11 $$\frac{F}{P}=2\pi^{1/4}{\left(\frac{w}{E^{*}R}\right)}^{1/2}{\left(\frac{P}{E^{*}R}\right)}^{-3/4}.$$

However, it is also noted that the *P*_2_=0 condition does not coincide with the maximum value of tangential force during complete stick.

### 3.3 Partial slip

After the maximum value of the tangential force for complete stick (defined as *F*_1_) is attained, solutions without slip are no longer possible. One possibility is complete slip (i.e. sliding) with the frictional shear stress (*τ*_S_) constant along the interface in the contact region, i.e.

3.12 $$F_{S}=2\tau_{S}a.$$

It is noted that depending on the relative values of *w* and *τ*_S_*R*, this value of *F*_S_ could be greater than the maximum value attained during stick. If so, there would also exist a finite range of the tangential force, i.e. *F*_1_<*F*<*F*_S_, for which neither of these two solutions exist.

We propose here that, irrespective of whether or not *F*_S_ is greater than *F*_1_, the contact zone can consist of an inner portion of stick −*d*<*x*<*d* surrounded by two slip zones. This assumption is similar to that used by Cattaneo [2] and Mindlin [3], in which the effect of adhesion was not included. In those analyses, any non-zero value of the tangential force exhibited a central contact region surrounded by two slip regions which satisfy Amontons–Coulomb friction. As the tangential force increases and becomes equal to the coefficient of friction multiplied by the normal force, the stick zone continuously shrinks to zero and complete sliding commences. Sari *et al.* [14] modified this formulation by including the effect of adhesion on the length of the contact region but not on the length of the stick region. A constant frictional shear stress in the slip regions was assumed.

In the present problem, because adhesion is broken in the slip zones, the contact zone is given by the Hertz equation (3.2) and in the slip zones there is a constant friction stress *τ*_S_. In the stick zone, however, adhesion has not been broken. Thus, the situation at *x*=±*d* is one of adhesion except that now *K*_*I*_=0. Consequently, the shear stress is written as [20]

3.13 $$\tau (x)=\left\{\begin{array}{cc} \frac{C_{0}}{\sqrt{d^{2}-x^{2}}}+\frac{2\tau_{S}}{\pi }\tan^{-1}\sqrt{\frac{a^{2}-d^{2}}{d^{2}-x^{2}}}, & -d<x<d,\\ \tau_{S}, & d<|x|<a. \end{array}\right.$$

The half-width (*d*) of the stick region can now be determined by setting *K*_II_ equal to its critical value, i.e.

3.14 $$K_{\mathrm{II}}=\lim_{x\to a}\sqrt{2\pi (a-x)}\tau (x)=\sqrt{2wE^{*}}$$

which leads to

3.15 $$C_{0}=\sqrt{\frac{2dE^{*}w}{\pi }}.$$

The resultant of the shear stresses is equal to the applied tangential load, i.e.

3.16 $$F=\int_{-a}^{a}\tau \text{d}x,\;\Rightarrow \;\frac{F}{2\tau_{S}a}=\sqrt{1-{\left(\frac{d}{a}\right)}^{2}}+\alpha \sqrt{\frac{d}{a}},$$

where

3.17 $$\alpha =\sqrt{\frac{\pi wE^{*}}{2\tau_{S}^{2}a}}.$$

Now the contact half-width *a* is determined by Hertz contact (i.e. equation (3.1) with *P*=*P*_1_) and the ratio of tangential to normal force can finally be written as

3.18 $$\frac{F}{P}=\frac{2(\tau_{S}/E^{*})(a/R)}{\left(P/E^{*}R\right)}\sqrt{1-{\left(d/a\right)}^{2}}+\alpha \sqrt{d/a},$$

where now *α* can be written as

3.19 $$\alpha =\frac{\pi^{3/4}{\left(w/E^{*}R\right)}^{1/2}}{2{\left(P/E^{*}R\right)}^{1/4}(\tau_{S}/E^{*})}.$$

In summary, for partial slip the contact half-width *a* is determined by Hertz contact (equation (3.2) with *P*=*P*_1_). Then equation (3.18) provides a simple algebraic relationship between the stick zone half-width *d* and the tangential force *F*.

## 4. Results and discussion

**Figure 2.**
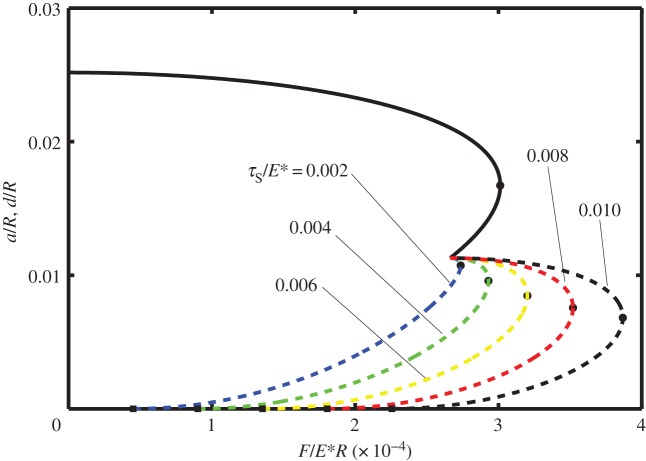
Dimensionless contact half-width (*a*/*R*) (solid lines) and stick half-width (*d*/*R*) (dashed lines) versus dimensionless tangential force (*F*/E*R) for the work of adhesion *w*/E*R=10^−6^ and with normal force *P*/E*R=0.0001, for various values of the shear stress (*τ*_S_/E*=0.002, 0.004, 0.006, 0.008 and 0.010).

**Figure 3.**
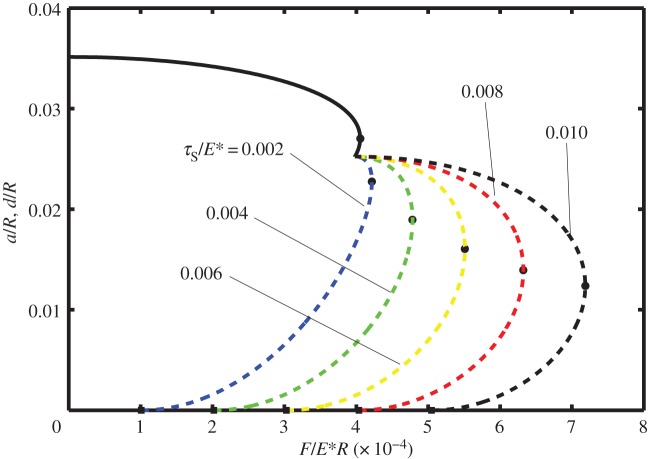
Dimensionless contact half-width (*a*/*R*) (solid lines) and stick half-width (*d*/*R*) (dashed lines) versus dimensionless tangential force (*F*/E*R) for the work of adhesion *w*/E*R=10^−6^ and with normal force *P*/E*R=0.0005, for various values of the shear stress (*τ*_S_/E*=0.002, 0.004, 0.006, 0.008 and 0.010).

**Figure 4.**
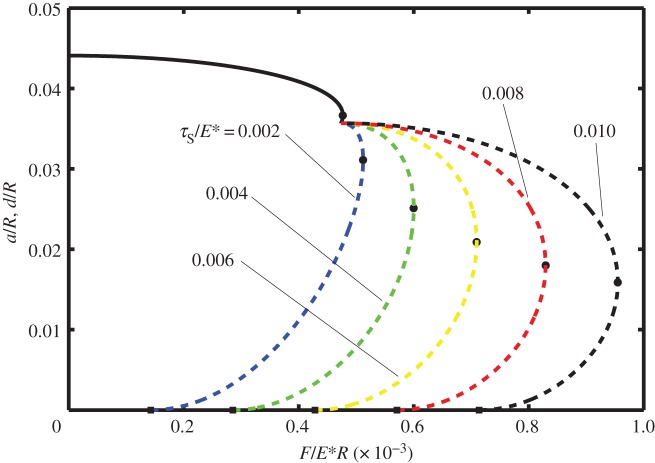
Dimensionless contact half-width (*a*/*R*) (solid lines) and stick half-width (*d*/*R*) (dashed lines) versus dimensionless tangential force (*F*/E*R) for the work of adhesion *w*/E*R=10^−6^ and with normal force *P*/E*R=0.0010, for various values of the shear stress (*τ*_S_/E*=0.002, 0.004, 0.006, 0.008 and 0.010).

In figures 2–4 it is shown how the non-dimensional contact half-width (*a*/*R*) and the stick zone half-width (*d*/*R*) vary with the dimensionless tangential force (*F*/E*R). All of these figures are for the dimensionless work of adhesion *w*/E*R=10^−6^ and for various values of the dimensionless shear stress *τ*_S_/E*=0.002, 0.004, 0.006, 0.008 and 0.010. Figures 2–4 correspond to different values of the dimensionless normal force, i.e. *P*/E*R=0.0001, 0.0050 and 0.0010, respectively. The solid lines correspond to *a*/*R* and are independent of the shear stress, whereas the dashed lines correspond to *d*/*R* and depend upon the shear stress.

Note that in each of these figures, there is a local maximum of the tangential force as a function of *a*/*R* (defined as *F*_1_ and shown in the figures with a small circle). Although there are mathematical solutions to equation (3.9) which correspond to smaller values of *a*/*R*, that branch of the curve is expected to be unstable. The smallest possible value of *a*/*R* corresponds to *P*_2_=0 and hence to Hertz contact. Smaller values of *a*/*R* are prohibited as these would lead to compressive values of *P*_2_ and the consequential overlap of the surfaces at the contact boundary.

Also note that if partial slip exists, the value of *d*/*R* is expected to depend on the shear stress in the slip zone as well as on the work of adhesion. Thus at the minimum (unstable) value of *a*/*R*, there are five curves for *d*/*R*, each corresponding to a different value of *τ*_S_/E*. As expected, the larger values of *τ*_S_/E* correspond to larger values of the tangential force. Furthermore, there is a maximum value of tangential force (which will be referred to as *F*_2_) at which the partial slip solution no longer exists. Greater values of tangential force are not possible and complete sliding is expected; the values of tangential force for complete sliding correspond to *d*/*R*=0 and are each shown by a square symbol in these figures.

**Figure 5.**
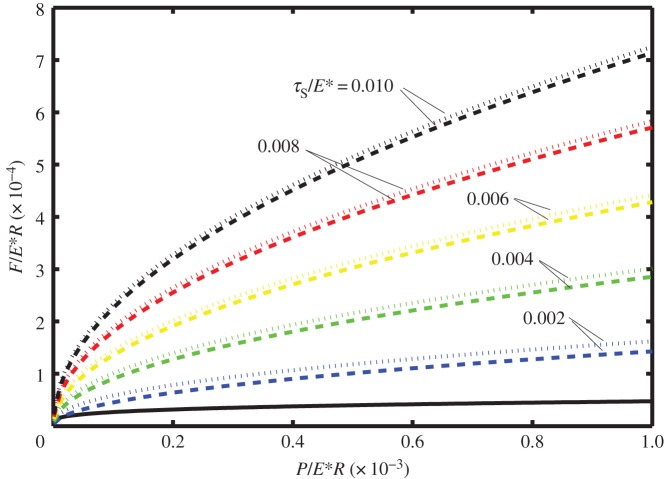
The dimensionless force *F*_1_ (maximum value of *F* for complete stick; solid line), *F*_2_ (maximum value of *F* for partial slip, dotted lines) and *F*_S_ (value of *F* for complete sliding; dashed lines) versus dimensionless normal force (*P*/E*R) for different values of the shear stress (*τ*_S_/E*=0.002, 0.004, 0.006, 0.008 and 0.010) and with the work of adhesion *w*/E*R=10^−8^.

**Figure 6.**
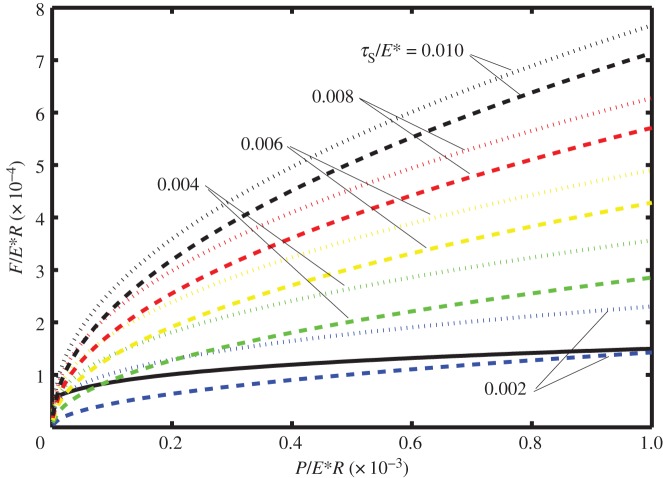
The dimensionless force *F*_1_ (maximum value of *F* for complete stick; solid line), *F*_2_ (maximum value of *F* for partial slip; dotted lines) and *F*_S_ (value of *F* for complete sliding; dashed lines) versus dimensionless normal force (*P*/E*R) for different values of the shear stress (*τ*_S_/E*=0.002, 0.004, 0.006, 0.008 and 0.010) and with the work of adhesion *w*/E*R=10^−7^.

**Figure 7.**
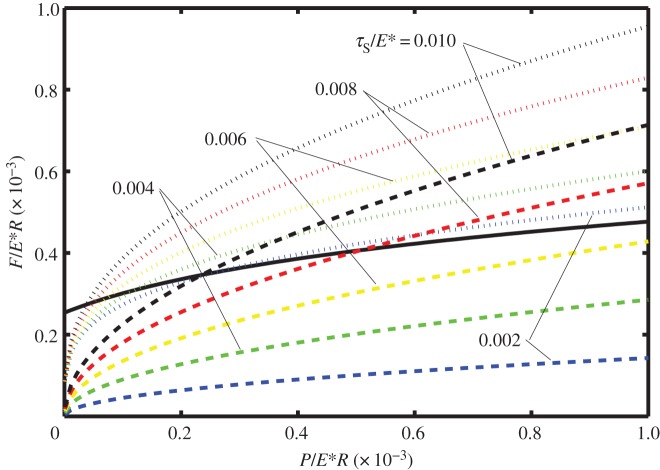
The dimensionless force *F*_1_ (maximum value of *F* for complete stick; solid line), *F*_2_ (maximum value of *F* for partial slip; dotted lines) and *F*_S_ (value of *F* for complete sliding; dashed lines) versus dimensionless normal force (*P*/E*R) for different values of the shear stress (*τ*_S_/E*=0.002, 0.004, 0.006, 0.008 and 0.010) and with the work of adhesion *w*/E*R=10^−6^.

Now consider figures 5–7 in which the dimensionless forces *F*_1_ (maximum value of *F* for complete stick), *F*_2_ (maximum value of *F* for partial slip) and *F*_S_ (value of *F* for complete sliding) are shown versus dimensionless normal force for different values of the dimensionless shear stress (*τ*_S_/E*=0.002, 0.004, 0.006, 0.008 and 0.010). There is only one curve for *F*_1_ because its value is independent of the shear stress. Also note that due to the effect of adhesion, the curve for *F*_1_ does not start at the origin, whereas each of the *F*_2_ and *F*_S_ curves do initiate at the origin. Each of these figures corresponds to a different value of the dimensionless work of adhesion, i.e. *w*/E*R=10^−8^, 10^−7^ and 10^−6^ for figures 5–7, respectively.

Because figure 5 has a relatively low value of the work of adhesion, the curve for *F*_1_ is considerably lower than are those for *F*_2_ and *F*_S_. Also note that for each value of the shear stress, the curves for *F*_2_ and *F*_S_ are close to each other with *F*_2_ slightly greater than *F*_S_. Both of these trends are due to the small effect of the work of adhesion. Because the termination of partial slip (*F*_2_) is associated with static friction whereas *F*_S_ is associated with sliding friction, the difference between the two is attributed to the difference between static and sliding friction.

Figure 7 has the highest value of the work of adhesion. It is observed that for each value of the shear stress, the curves for *F*_2_ and *F*_S_ deviate significantly from each other with *F*_2_ greater than *F*_S_. Also note that *F*_1_ can be greater than *F*_2_ for sufficiently small combinations of the normal load and shear stress. Both of these trends are due to a large effect of the work of adhesion. In those cases for which *F*_1_ is greater than *F*_2_, the partial slip region does not exist and the termination of complete stick (*F*_1_) is associated with static friction, whereas *F*_S_ is associated with sliding friction; the difference between the two is attributed to the difference between static and sliding friction.

It is noted that if both materials are metals with a composite Young’s modulus (E*) of 100 GPa and a work of adhesion (*w*) of 1 J m^−2^, then the cylinder radius (*R*) corresponding to *w*/E*R=10^−6^ is 10 μm. If *w*/E*R=10^−8^, then the corresponding radius is 1 mm. Similarly for a polymer with composite modulus (E*) of 1 GPa and a work of adhesion (*w*) of 10 mJ m^−2^, then the corresponding cylinder radius also varies from 10 μm to 1 mm as *w*/E*R varies from 10^−6^ to 10^−8^.

## 5. Conclusion

The plane strain linear elastic problem of a cylinder pressed against an elastic half-space has been investigated, with the effect of adhesion included through the use of surface energy. While the compressive normal force is held constant, a tangential force is applied and gradually increased. The contact is characterized by complete stick up to a critical value of the tangential force. At that critical value, there is a sudden transition either directly to complete sliding or to a partial slip state in which two slip regions surround a central stick region. In the latter case, at a finite value of the stick zone width a second critical condition exists at which there is a sudden transition from partial slip to complete sliding. The results show that the sliding friction force is always less than the maximum of either the complete stick or partial slip tangential forces. This behaviour is plotted for different dimensionless values of the work of adhesion, the shear stress during slip/sliding and the initial compressive load.
